# An ethnobotanical study of medicinal plants used in Kilte Awulaelo District, Tigray Region of Ethiopia

**DOI:** 10.1186/1746-4269-9-65

**Published:** 2013-09-08

**Authors:** Abraha Teklay, Balcha Abera, Mirutse Giday

**Affiliations:** 1 Department of Biology, Jimma University, Jimma, Ethiopia; 2 Aklilu Lemma Institute of Pathobiology, Addis Ababa University, Addis Ababa, Ethiopia

**Keywords:** Ethnobotany, Medicinal plants, Kilte Awulaelo District, Eastern Tigray, Ethiopia

## Abstract

**Background:**

The Ethiopian people have been dependent on traditional medicine, mainly medicinal plants, from time immemorial for control of human and animal health problems, and they still remain to be largely dependent on the practice. The purpose of the current study was to conduct ethnobotanical study to document medicinal plants used to treat diseases of human and domestic animals in Kilte Awulaelo District in the Tigray Region of Ethiopia.

**Methods:**

Ethnobotanical data were collected between July and September 2011 through semi-structured interviews, ranking exercises and field observations. For the interviews, 72 knowledgeable informants were sampled using purposive sampling method. For the different ranking exercises, key informants were identified with the help of elders and local administrators from informants that were already involved in the interviews.

**Results:**

The study revealed 114 medicinal plant species belonging to 100 genera and 53 families. The plants were used to treat 47 human and 19 livestock diseases. Of the species, the majority (74%) were obtained from the wild. Herbs were the most utilized plants, accounting for 44% of the species, followed by shrubs (29%). Leaf was the most commonly used plant part accounting for 42.98% of the plants, followed by roots (25.73%). Preference ranking exercise on selected plants used against abdominal pain indicated the highest preference of people for *Solanum marginatum*. Direct matrix ranking showed *Cordia africana* as the most preferred multipurpose plant in the community. Preference ranking of selected scarce medicinal plants indicated *Myrica salicifolia* as the most scarce species, followed by *Boscia salicifolia* and *Acokanthera schimperi*. According to priority ranking, drought was identified as the most destructive factor of medicinal plants, followed by overgrazing and firewood collection.

**Conclusion:**

Medicinal plants are still playing significant role in the management of various human and livestock diseases in the study area with herbs taking the lead in the number of plants used in the preparation of remedies, which may be an indication of their relatively better abundance as compared to other life forms. Recurrent drought was reported to have seriously threatened medicinal plant resources in the District. Awareness is thus needed be raised among local people on sustainable utilization and management of plant resources. *Ex situ* and *in situ* conservation measures should be taken to protect the medicinal plants of the District from further destruction and special attention should be given to the medicinal plants that were indicated by preference ranking exercise as the most threatened ones.

## Background

About 80% of the Ethiopian population and 90% of livestock still depend on traditional medicinal to fight a number of diseases [1,2]. The reliance on medicinal plants is partly owing to the high cost of modern drugs, inaccessibility of modern health institutions and due to cultural acceptability of the system [3-5]. However, as time goes on, the traditional knowledge and the associated plants in the country are gradually being depleted for reasons mainly attributed to environmental degradation and deforestation, which in turn brought about the loss of some important medicinal plants [4,6]. On the other hand, documentation work related to traditional medical knowledge in the country still remains at minimum level calling for conduct of more ethnobotanical studies.

The people of Tigray Region, in general, and Kilte Awulaelo District, in particular, are also expected to have rich knowledge on traditional medicine involving medicinal plants. Such knowledge is, however, currently being threatened, as it is happening elsewhere in the country, due to environmental degradation and deforestation. On the other hand, published reports indicate that only few ethnobotanical studies have been conducted in Tigray to properly document the use of medicinal plants [7-10]. The studies conducted in the districts of Alamata [7], Enderta [8], Hawzen [9], and Asgede Tsimbila [10] documented 25, 27, 33 and 68 medicinal plants, respectively. However, no such study has far been conducted in Kilte Awulaelo District. The purpose of the current study was, therefore, to gather and document information on the use of medicinal plants by people in Kilte Awulaelo District, in Tigray Region of in Ethiopia, to manage diseases of humans and domestic animals. The study was expected to play a role in prioritizing medicinal plants in the District for further evaluation and conservation.

## Materials and methods

### Description of study district and people

**Figure 1:**
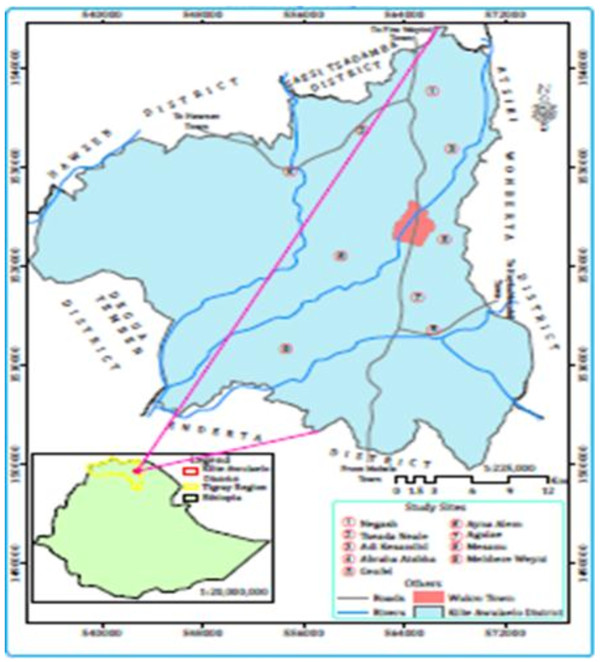
Map of the Kilte Awlaelo District (modified based on GIS of Ethiopia); red and black dots showing selected study kebeles.

Kilte Awulaelo District is located at 825 km north of the capital Addis Ababa, in the Eastern Zone of Tigray Regional State, Northern Ethiopia. It shares borders with the districts of Howzien and Sease Tsadamba in the north, Atsbi Womberta in the east, Douga Tembien in the west and Enderta in the South (Figure 1). The District is composed of 18 kebeles. Kebele is the smallest administrative unit in Ethiopia. Altitude in the District ranges between 1900 and 2460 meters above sea level. It covers an area of 101,758 hectares, of which 21,620 hectares are farmlands, 7,930.85 hectares are grazing areas, 44,134 hectares are enclosure areas and 28,073.15 hectares are occupied by hills and residential areas [11], Kilte Awulaelo Plan and Finance Office, unpublished data, 2010.

According to 2001-2010 rainfall data, the District has a high rainfall distribution between July and August and a smaller rainfall between March and June and in September. The mean monthly rainfall and mean annual rainfall of the District are 50.14 mm and 601.68 mm, respectively [National Metrological Service Agency, Mekelle Branch Office, unpublished data].

Tigrigna, an official language in Tigray Region, is the language spoken by the people residing in Kilte Awlaelo District. The language belongs to the Semitic language family. Based on the population census of 2007 by the Ethiopian Central Statistical Agency [12], the total population of the District is 111,546, of which 48.89% are males and 51.11% are females.

The people in District mainly cultivate barely, wheat, teff, bean, pea, maize and sorghum. Of the domestic animals raised in the District, poultry has the population, estimated to be 62,610 heads, followed by cattle (61,864), shoats (56,042), honey bees (54,217) [11], Kilte Awulaelo Plan and Finance Office, unpublished data, 2010.

Malaria, upper respiratory problems, skin infection, infestation of intestinal parasites, pneumonia, soft tissue injury, gastritis, diarrheal, arthritis and eye diseases have been reported as the ten most common diseases in the District. In 2010, there were five functional health centres in the District where the kebeles Agulae, Negash, Beati Akor, Tsige Reda and Abraha Atsbha had one each [Kilte Awulaelo Health Office, unpublished data, 2010].

### Selection of study kebeles and informants

Ethnobotanical data were collected between July and September 2011 from nine kebeles that were purposively selected with the help of elders and local authorities of the District based on better availability of traditional healers and knowledgeable people. The kebeles were Ayne Alem, Negash, Tseada Neale, Agulae, Abraha Atsbha, Adi Kesandid, Genfel, Mahbere Weyni, and Mesanu.

For the interview, 72 healers and knowledgeable informants (eight from each sampled kebele) were selected using purposive sampling method [13], of which 61 were males and 11 were females. The informants selected from each sampled kebele were the most knowledgeable ones as suggested by respective kebele elders and administrators who participated in the selection process. The ages of the informants ranged between 20 and 82 years. Three key informant groups (one group containing nine individuals) were respectively involved in three different ranking exercises (two preference ranking exercises and one direct matrix ranking exercise). The key informants were selected from the already interviewed informants with help of elders and local administrators.

### Ethnobotanical data collection

Ethnobotanical data were collected through semi-structured interviews and observations by following standard methods [13,14]. Series of individual interviews were carried out to gather information regarding local names of plants used, their threats and management, part(s) used, preparations methods, routes of remedy administration, diseases treated and side effects of remedies. The same method was also used to collect data on habit, habitat, marketability and conservation status of the reported medicinal plants. Interviews were conducted using Tigrigna, language that is spoken by the people in the study District. For each reported plant species, specimen was collected, pressed, dried, and identified and voucher was kept at Jimma University Herbarium. Field observations were also used to record habit and habitat of each medicinal plant with the assistance of local guides and interviewed informants. The study was ethically approved (before its commencement) by the Graduate Program Evaluation Committee of the College of Natural Science, Jimma University.

### Data analysis

The data were summarized using Microsoft Office Excel 2007 computer programme. Descriptive statistical methods were employed to analyze and summarize the ethnobotanical data.

Preference ranking exercise [13] was conducted by nine key informants on six medicinal plants used to treat abdominal pain in the District. Abdominal pain was the disease against which the highest number o f medicinal plants was prescribed by informants. The plants used in such exercise were short-listed by the key informants following group discussion on their importance to manage abdominal pain. The informants were given the plants and asked to arrange them based on their personal level of efficacy. Medicinal plant that was believed to be the most effective was given the highest value, i.e. 6, and the one with the least effectiveness a value of 1 and rank was determined based on the total score of each species.

Preference (priority) ranking exercise [13] was conducted by nine key informants on six medicinal plants, shorted-listed by the same, to rank them based on informants’ perceived level of threat. A value of 6 was given for the most scarce medicinal plant and 1 for the least scarce ones, and scores of each species were finally summed and ranked.

Direct matrix ranking exercise [13] was employed on seven medicinal plants that were most frequently reported as multipurpose medicinal plants in the study District. A group of nine key informants were asked to rank the plants with different uses (including their use as medicinal plants) through discussion based on their perceived level of usefulness using a numerical scale (0 for no value, 1 for lowest value and 7 the highest value). Values assigned for each plant were added together to determine its rank. Medicinal plant having the highest values is ranked first, an indication of its highest level of threat.

## Results

### Medicinal plants reported

The study conducted in Kilte Awlaelo District recorded 114 medicinal plant species (Tables 1, 2 and 3). The species belonged to 100 genera and 53 families. The family Lamiaceae was represented 9% of the reported species, followed by Fabaceae (8%), Solanaceae (7%), Euphorbiaceae (6%) and Asteraceae (4%). Of the total plants, 44% were herbs, 29% were shrubs, 19% were trees and 8% were climbers.

**Table 1:** Medicinal plants used to treat both human and livestock diseases

**Scientific name**	**Family name**	**Voucher No.**	**Tigrigna name**	**Habit**	**Part used**	^**1**^**Used for**	**Preparation and application**
*Acacia etbaica* Schweinf.	Fabaceae	AT00606	Seraw	Tree	Leaf	Swelling	Crush and mix with latex of *Euphorbia abyssinica* and rub the paste on the affected part
					Leaf	Eye infection (livestock)	Chew and spit on to the affected eye
					Stem	Ring worm	Place it on fire and apply liquid on the affected part
					Stem	Haemorrhoids	Burn and place it on the affected part
					Leaf	Itching/ scabies	Crush and rub
					Stem	Anthrax (both)	Burn and place it on the affected part
					Leaf	Fire burn	Crushed and apply on the burn
*Achyranthes aspera* L.	Amaranthaceae	AT00654	Muchele	Herb	Leaf	Tonsillitis	Crush it alone or with leaves of *Cucumis ficifolius* and place it on shaved head of child
					Root	Eye infection (livestock)	Chew and spit the liquid onto the infected eye
					Root	Anthrax (both)	Crush it with roots of *Solanum incanum* and whole part of *Hypoestes forskaolii*, add water and drink with cup of glass
					Root	Urine retention	Tie around the sex organ
					Leaf	Eye infection	Boil with leaves of *Eucalyptus globulus*, *Withania somnifera* and *Zehneria scabra* in water and inhale the vapour
					Root	Snake bite	Chew and swallow the fluid
					Leaf	Wound	Crush and place on the wound
					Root	Babesia (livestock)	Crush and apply into the nose
					Leaf	Wound/sore	Crush and rub the past on the wound
					Root	Paralysis	Crush, dry and put it on fire for fumigation
*Aloe megalacantha* Bark.	Aloaceae	AT00707	Ere	Shrub	Root	Dislocated bone (livestock)	Tie it on the ear
					Latex	External wound	Cut a leaf and spread latex on wound until healed
					Leaf	Malaria, amoeba	Crush leaves and squeeze juice, filter and drink
					Latex	Ascariasis	Squeeze latex, filter and drink
					Leaf	Abdominal pain	Crush leaves, filter and drunk the fluid
					Latex	Ticks infestation (livestock)	Cut leaf and apply latex on skin
					Leaf	Evil eye	Place leaf on fire and fumigate
					Root	Impotence	Crush root and mix butter and smear it on the penis
					Whole	Snake bite (both)	Crush the part and drink the juice
					Root	Urine retention	Tie it around the sex organ
*Anethum graveolens* L.	Apiaceae	AT00677	Shilan	Herb	Whole	Urine retention (both)	Crush, filter and drink the fluid
							Boil it in water and drink the fluid
*Calotropis procera* (Aiton) W.T. Aiton	Asclepiadaceae	AT00612	Ginda	Shrub	Leaf & latex	External wound	Crush it with leaves of *Ficus palmata* and smear paste on affected part until healed
					Latex	Tinea capitis	Cut leaf or stem and smear latex on affected part until healed
					Leaf	Itching/skin rash	Burn and grind leaves and spread on skin with butter
					Latex	Swelling (livestock)	Apply latex on affected part
*Calpurnia aurea* (Ait.) Benth.	Fabaceae	AT00614	Hitsawts	Tree	Leaf	Lice and ticks infestation (livestock)	Crush and apply paste on skin
					Seed	Diarrheal	Grind part, mix it with milk product locally called (mancheba) and drink
					Leaf	Abdominal pain	Crush, filter and drink the fluid
					Leaf	Herpes zoster	Dry the leaves and mix them with leaves of *Datura stramonium* and leaves of *Clutia abyssinic*a, grind them and make past with butter and apply on affected part
					Seed	Haemorrhoids	Grind and mix it with honey and milk and eat it
					Leaf	Tinea capitis	Crush and spread paste on the affected part
*Chenopodium murale* L.	Chenopodiaceae	AT00650	Hamli qiweo	Herb	Leaf	Swelling (both)	Crush part and apply on the affected part
					Leaf	Fire burn	Burn leaves in oven, grind, mix with butter and smear paste on affected part
					Leaf	Anthrax (human)	Placing around fire then after rubbing by the leaf to the affected part
					Leaf	External wound	Crush the part and apply it on the wound
					Leaf	Dandruff	Crush and smear on affected part
*Clematis hirsuta* Perr. & Guill.	Ranunculaceae	AT00680	Hareg	Climber	Root	Anthrax (both)	Crush roots with butter and apply the paste
					Leaf	Herpes zoster	Burn leaves in oven with leaves of *Dodonaea angustifolia*, grind, mix with butter and apply on the affected part
*Clerodendrum myricoides* (Hochst.) R.Br. ex Vatke	Lamiaceae	AT00643	Shiwha	Shrub	Root	Urine retention (both)	Crushed, add water, filter and drink
					Stem	Snake bite	burn stem and apply it on the affected part while hot
					Leaf	Tinea captis	Burn leaves in oven, grind, add butter and rub it on affected part
					Root	Evil sprit	Crush by mixing with roots of *Withania somnifera*, *Carissa spinarum*, *Jasminum gratissimum* and *Maytenus senegalensis*, put it on fire for fumigation
*Clutia abyssinica* Jaub. & Spach.	Euphorbiaceae	AT00659	Tewshealalito	Shrub	Leaf	Tinea capitis	Crush leaves and rub the paste on affected part or dry the crushed leaves, add butter and apply
					Leaf	Ring worm	Rubbing the affected part by the leaf
					Leaf	Internal parasites infection (livestock)	Crushed and drunk the fluid
					Leaf	Herpes zoster	Dry and mix leaves with dried leaves of *Calpurnia aurea* and *Datura stramonium*, grind, add butter and spread paste on affected part
					Leaf & bark	Black spider bite	Crush, filter and drink
					Leaf	Leshimaniasis	Crush and apply paste on affected part
*Cucumis ficifolius* A.Rich.	Cucurbitaceae	AT00642	Ramboambo	Herb	Leaf	Anthrax (both)	Crush the part with leaves of *Dyschoriste radicans* , mix it with honey and placed it on affected part
					Root	Snake, scorpion and black spider bite	Grind the part, mix with honey and eat it
					Fruit	Wound/sore	Apply fruit juice on the affected part
					Whole	Jaundice/ hepatitis	Crush, add water, filter and drink or Chew and swallow the product
					Leaf	Tonsillitis	Mix the leaves of the plant with leaves of *Achyranthes aspera*, crush and place the paste on shaved part of head of a sick child
					Root	Toothache	Chew part with the diseased teeth
					Root	Joint pain	Crush, filter and drink the fluid
					Root	Abdominal pain (both)	Mix root of the plant with bark of *Croton macrostachyus*, dry the paste, mix it with butter and drink it or chew the product and drink the fluid
					Root	Vomiting	Crush and drink the fluid
*Cyphostema adenocaule* (Steud. ex A.Rich.)	Vitaceae	AT00681	Aserkuca asergundi	Climber	Root	Snake bite (both)	Crush, filter and drunk the fluid
					Root	Snake venation (repulsion)	Tie it on the body
*Cyphostemma junceum* (Webb) Desc. Ex Wild & R.B.Drumm.	Vitaceae	AT00686	Etse zewye	Herb	Root bark	Snake bite (both)	Crush and eat the paste with honey
					Whole	Toothache	Chew and swallow the juice
					Whole	Spider bite	Chew and swallow fluid
					Leaf	Evil eye	Place part on fire for fumigation
*Datura stramonium* L.	Solanaceae	AT00672	Mestenagr	Herb	Leaf	Itching/ scabies	Crush and apply on affected part
					Leaf	External wound	Crush and apply on affected part
					Seed	Toothache	Place it on fire and inhale the smoke through mouth
					Leaf	Herpes zoster	Dried leaves of the plant and *Calpurnia aurea* and *Clutia abyssinica* are ground mixed powder with butter and apply on affected part
					Leaf	Wound/sore	Crush and apply on affected part
					Leaf	Anthrax (livestock)	Crush by mixing with leaves of *Solanum mariginatum* and *Malva verticillata* and apply paste on affected part
*Dodonia angustifolia* L.f.	Sapindaceae	AT00610	Tahses	Tree	Leaf	Herpes zoster	Dry the leaf of the plant alone or mixed with the leaf of *Clematis hirsuta* on hot stove, grind, add butter and rub affected part
					Seed	Malaria	Grind and eat it with honey
					Leaf	Dislocated bone (livestock)	Crush and apply on damaged part
					Leaf	Eye infection	Crush and apply droplets into the infected eye
					Leaf	Fire burn	Dry it in oven, grind, and butter sulphur and spread it on the affected part
*Echinops Kebericho* Mesfin	Asteraceae	AT00685	Dander	Herb	Root	Dislocated bone (livestock)	Tie it on damaged part
					Stem	Haemorrhoids	Place a burned stem and apply it on affected while hot
*Euclea racemosa* Murr. subsp. *schimperi* (A.DC.) F. White	Ebenaceae	AT00611	Keleaw	Shrub	Whole	Evil eye	Crush and tie powder around the neck
					Root bark	Snake bite (both)	Crush, add water and drunk the fluid
					Root	Paralysis	Crush and drink juice with milk
					Root	Black spider bite	Chew and swallow the fluid
					Root bark	Abdominal pain	Boil it in water and drink the fluid
					Root	Toothache	Chew part with the affect tooth
					Root	Amoeba	Remove bark of the root, boil it and drink the fluid with mancheba, milk product
*Euphorbia abyssinica* J.F.Gmel.	Euphorbiaceae	AT00706	Kulqual	Tree	Latex	Ascariasis	Mix part with locally made beer and drink it or mix it enjera (local food) and eat it
					Latex	Abdominal pain	Mix it with tihni (made from flour of roasted barley) and eat it
					Flower	External wound	Crush, mix with honey and apply it on affected part
					Latex	Leprosy	Smear latex on affected part
					Latex	Swelling (both)	Smear latex on affected part
*Euphorbia petitiana* A.Rich.	Euphorbiaceae	AT00687	Hindukduk	Herb	Latex	Leshimaniasis	Rub leaf on affected part until cure
					Root	Dislocated bone (livestock)	Tie around the damage part
*Euphorbia tirucali* L.	Euphorbiaceae	AT00682	Kinchib	Shrub	Latex	Skin haemorrhoids	Apply on affected part
*Galium boreo-aethiopicum* Puff	Rubiaceae	AT00621	Mendef adgi	Herb	Root	Babesia (livestock)	Crush and apply droplets through the nose
					Root	Toothache	Chew root with affected tooth
					Root	Evil eye	Placing it on fire for fumigation
					Root	Abdominal pain	Chew and swallow fluid
					Latex	Ring worm	Apply latex on affected part
					Latex	Swelling (livestock)	Smear latex on swollen part
*Gomphocarpus purpurascens* A.Rich.	Asclepiadaceae	AT00690	Tseba dimu	Herb	Root	Abdominal pain	Chew and swallow the fluid
					Whole	Haemorrhoids	Crush and apply on affected part
					Root	Wound (livestock)	Crush and apply on affected part
					Root	Toothache	Mix part with honey and chew
					Leaf	Michi	Place on fire for fumigation
*Hypoestes forskaolii* (Vahl) Roem. & Schult.	Acanthaceae	AT00603	Girbia	Herb	Root	Babesia (livestock)	Crush, mix with honey and eat
					Whole	Anthrax	Crushed it alone or by mixing with seeds of *Lepidium sativum*, roots of *Solanum incanum*, *Achyranthes aspera* and *Verbascum sinaiticum*, filter and drink the fluid
					Root	Abdominal pain	Chew and swallow the fluid
					Leaf	Wound/sore	Crush apply on affected part
					Root	Ascariasis	Boiling in milk with leaves of *Lantana trifolia* and drunk
					Root	Cough	Place it on fire for fumigation
*Hypericum annulatum* Moris.	Hypericaceae	AT00668	Aklti	Herb	Leaf	Eye infection (both)	Dry, Grind, add butter and apply on affected part
*Justicia schimperiana* (Hochst. ex Nees) T.Anders.	Acanthaceae	AT00632	Shimieya	Shrub	Leaf	Dysentery	Crush, add water and drink
					Leaf	Jaundice	Crush and eat it with enjera (local food) or add milk and drink it
					Leaf	Wound	Boiling with roots of *Withania somnifera* and washing
					Leaf	Arthritis	Boil it in water and wash body with it
*Laggera tomentosa* (Sch.Bip ex A.Rich.)	Asteraceae	AT00679	Kash koshe	Shrub	Leaf	Leeches infestation (livestock)	Crush and add juice through the nose
					Leaf	Ring worm	Rub it on affected part
*Lepidium sativum* L.	Brassicaceae	AT00708	Shimfa	Herb	Seed	Wound/sore	Crush seeds leaves of *Dyschoriste radicans* and bulb of *Allium sativum* and tie on affected part
					Seed	Itching/scabies	Crush seeds with leaves of *Rumex nervosus* and *Withania somnifera* and bulb of *Allium sativum*, sock it in water and wash body with it
					Seed	Swelling (both)	Crush and apply it on affected part
					Seed	Abdominal pain	Crush, add water and drink
					Seed	Anthrax (both)	Crush it by mixing it with whole part of *Hypoestes forskaolii*, roots of *Solanum incanum* and *Verbascum sinaiticum*, filter and drink the fluid
					Stem	Haemorrhoids	Burn it on fire add apply it on affected part while hot
					Leaf	Michi	Crush it with bulbs of *Allium sativum* and eat or rub it on the skin
*Malva verticillata* L.	Malvaceae	AT00625	Enkiaftha	Herb	Leaf	External wound	Crush and rub apply it on the affected part
					Leaf	Anthrax (both)	Crush it with leaves of *Datura stramonium* and *Solanum mariginatum* and apply it
*Melia azadrachta* L.	Meliaceae	AT00695	Limo, nim	Tree	Leaf	External wound (both)	Crush and apply it
*Opuntia ficus-indica* (L.) Miller.	Cactaceae	AT00713	Beles (kulqual bahri)	Shrub	Leaf	Anthrax	Place it on fire and apply it on affected part while hot
					Leaf	Lice or fleas infestation (livestock)	Crush, rub on skin
					Leaf	Dandruff	Crush and rub it on affected part
*Otostegia integrifolia* Benth.	Lamiaceae	AT00652	Chiendog	Shrub	Leaf	Ascariasis	Crush, filter and drink the fluid
					Root	Abdominal pain	Chew and swallow the fluid
					Leaf	Lice or fleas infestation (livestock)	Place it on fire for fumigation
*Premna oligotricha B*aker	Lamiaceae	AT00633	Sasa	Shrub	Whole	Synerosis celebralis (livestock)	Place on fire for fumigation
					Leaf	Toothache	Chew it with affected tooth
*Ricinus communis* L.	Euphorbiaceae	AT00688	Guile	Herb	Leaf	External wound (both)	Crush and apply it on the wound
*Rumex nepalensis* Spreng.	Polygonaceae	AT00618	Dengele, shembobata	Herb	Leaf	Ring worm	Rub it on affected part
					Root	Fire burn	Crush by mixing it with urine and apply it on damaged part
					Leaf	Tinea capitis	Mix it with fruit of *Citrus aurantifolia* and rub it affected part
*Schinus molle* L.	Anacardiaceae	AT00648	Tikur berbere	Tree	Leaf	Jaundice	Crush, filter and drink the fluid
					Leaf	Diarrheal	Crush, filter and drink the fluid
					Leaf	Bloating (livestock)	Crush and drink the fluid
					Leaf	Tonsillitis	Crush and drunk it with coffee
							Crush and apply it on shaved head of sick child
					Leaf	Michi	Crush, filter and drink the fluid
*Sida schimperiana* Hochst. ex A.Rich.	Malvaceae	AT00636	Tifraria	Shrub	Root	Rh disease	Crush, filter and drink a cup of fluid
					Root	Paralysis	Tie root around the affected part
					Root	Dislocated bone (livestock)	Tie it on tail of the affected animal
					Root	Abortion (livestock)	Tie it on tail of the animal
*Solanum hastifollium* Hochst. ex Dunal in DC.	Solanaceae	AT00641	Alalmo kalbi	Shrub	Root	Abortion (livestock)	Tie it on tail of the animal
					Root	Evil eye	Place it on fire for fumigation
					Leaf & fruit	Anthrax (livestock)	Crush and add honey and apply or squeeze it into affected part or crush, filter and drink
					Leaf	Tonsillitis	Crush by mixing it with leaves of *Solanum incanum* and place on shaved head of sick child
					Fruit	Ear diseases	Squeeze to produce juice, add goat butter and apply through the nose
					Leaf	Cellulitis	Crush and apply
					Root	Toothache	Chew with affected part
*Solanum incanum* L.	Solanaceae	AT00617	Niesheton engule	Shrub	Root	Abdominal pain	Chew and swallow the fluid
					Root bark	External wound infection	Dry, grind and apply on affected part
					Leaf	Tonsillitis	Crush by mixing with leaves of *Solanum hastifolium* and place it on the shaved head of sick child
					Root	Anthrax (both)	Crush by mixing with seeds of *Lepidium sativum*, whole part of *Hypoestes forskaolii*, roots of *Achyranthes aspera* and *Verbascum sinaiticum*, filter and drink fluid
							Crush and apply on affected part
*Solanum mariginatum* L.f.	Solanaceae	AT00716	Aby ungule	Shrub	Root	Abdominal pain	Chew and swallow the fluid or boil and drink the fluid
					Seed	External wound infection	Grind and apply on affected part
					Seed	Cough	Burn, grind, mix it with sugar or honey and swallow it
					Fruit	Breathing problem (livestock)	Crush apply through the nose
					Leaf	Anthrax (both)	Crush by mixing with leaves of *Datura stramonium* and *Malva verticillata* apply it on affected part or crush, filter and drink the fluid
					Root	Ascariasis	Crush by mixing with roots of *Zehneria scabra*, and *Verbena officinalis*, filter and drink the fluid
*Trigonella foenum-graecum* (Bunge) Gurke.	Fabaceae	AT00699	Abeake	Herb	Seed	Abdominal pain	Grind, add water and drink
					Seed	Swelling (both)	Grind by mixing with beans and rub paste on affected part
*Verbascum sinaiticum* Benth.	Scrophulariaceae	AT00634	Tirnake (handega)	Herb	Leaf	Fire burn	Crush and apply fluid on the burned skin
					Root	Tonsillitis	Crush, filter and drink with a cup
					Root	Evil eye	Place it on fire with sulphur for fumigation
					Root	Toothache	Chew it
					Root bark	Haemorrhoids	Crush, filter and drink
					Leaf	External wound	Crush and apply on affected part
					Root	Anthrax (livestock)	Crush with seeds of *Lepidium sativum*, roots of *Solanum incanum* and whole part of *Hypoestes forskaolii*, filter and drink the fluid
							Crush, mix with honey and eat it
					Root	Dislocated bone	Tie around the affected part
*Withania somnifera* (L.) Dunal	Solanaceae	AT00630	Agol	Herb	Leaf	Eye infection	Boil it in water by mixing with leaves of *Eucalyptus globulus*, roots of *Achyranthes aspera* and *Cynoglossum lanceolataum* and leaves of *Zehneria scabra* and inhale the vapour
					Whole	Evil eye	Crush by mixing with roots of *Carissa spinarum* and put it on for fumigation
					Whole	Michi	Soak in water by mixing with leaves of *Rumex nervosus* and juices of *Citrus aurantifolia* and wash body with it
							Boil by mixing with *Justicia schimperiana* and wash the body with it
							Place it on by mixing with leaves of *Zehneria scabra* and *Eucalyptus globulus* and fumigate
					Leaf	Itching/ scabies	Crush by mixing with leaves of *Rumex nervosus*, seeds of *Lepidium sativum* and bulbs of *Allium sativum*, soak it in water and wash affected part with it
					Root	Paralysis	Place it on fire for fumigation
					Root	Evil sprit	Crush by mixing with roots of *Clerodendrum myricoides*, *Carissa spinarum*, *Jasminum gratissimum* and *Maytenus senegalensis* and place it on fire for fumigation

^1^Unless indicated, the disease is that of human.

**Table 2:** Medicinal plants used to treat human diseases only

**Scientific name**	**Family name**	**Voucher No.**	**Tigrigna name**	**Habit**	**Part used**	**Used for**	**Preparation and application**
*Abutilon bidentatum* (Hochst.) A.Rich.	Malvaceae	AT00635	Neger negarito	Shrub	Leaf	Michi	Crush, filter and drink
					Leaf	Abdominal pain	Crush, filter and drink by adding milk
*Acacia abyssinica* Hochst.ex Benth.	Fabaceae	AT00700	Memona	Tree	Bark	Herpes zoster	Crush and apply on affected part
					Root	Evil eye	Crush and place on fire for fumigation
*Acokanthera schimperi* (A.DC.) Schweinf.	Apocynaceae	AT00649	Mebtie	Tree	Bark	Itching/ scabies	Boil it in water and wash body with it
*Ajuga integrifolia* Buch-Ham.	Lamiaceae	AT00675	Endifdif	Herb	Leaf	Tinea capitis	Crush and rub on the affected part
					Leaf	Ascariasis	Crush, filter and drink
					Leaf	Tap worm	Crush, filter and drink
					Leaf	Abdominal pain	Crush, filter and drink
*Allium sativum* L.	Alliaceae	AT00709	Tsaeda shingurti	Herb	Bulb	Wound/sore	Crush by mixing with leaves of *Dyschoriste radicans* and *Lepidium sativum* and tie on the affected part
					Bulb	Cough	Eat or smell the part or crush and eat it with honey
					Bulb	Paralysis	Crush and rub on body
					Bulb	Toothache	Chew with the affected tooth
					Bulb	Itching/scabies	Crush by mixing with leaves of *Rumex nervosus* and *Withania somnifera* and seeds of *Lepidium sativum*, soak it in water and wash body with it
					Bulb	Malaria	Crush and it alone or by mixing with seeds of *Lepidium sativum* and eat it
					Bulb	Amoeba	Grind and eat it with honey
					Bulb	Rabies	Eat the part
					Whole	Michi	Crush and apply the paste or place it on fire for fumigation
*Amorphophallus abyssinicus* (Rich.) N.E.Br.	Araceae	AT00637	Hambagita	Herb	Leaf	Tinea capitis	Crush and apply on affected part
*Argemone mexicana* L.	Papaveraceae	AT00615	Eshok tilian, medafe	Herb	Latex	External wound	Apply it on the affected part until cure
					Latex	Leshimaniasis	Apply it on affected part until cure
					Leaf	Tinea capitis	Crush and apply
*Artemisia abyssinica* Sch.Bip. ex A.Rich.	Asteraceae	AT00678	Chena baria	Herb	Whole	Evil eye	Mix with bulbs of *Allium sativum* and smell it
					Leaf	Michi	Crush by mixing with bulbs of *Allium sativum* and seeds of *Lepidium sativum*, add water and rub it on the skin
*Asparagus africanus* Lam.	Asparagaceae	AT00658	Kastanito	Climber	Root	Dislocated bone	Tie it on affected part
					Root	Evil eye	Place on fire for fumigation
					Root	Leshimaniasis	Crush, mix it with honey and apply on affected part
*Becium grandiflorum* (Lam.) Pic.Serm.	Lamiaceae	AT00609	Tebeb	Shrub	Root	Black spider bite	Chew and swallow the fluid
*Bidens camporum* (Hutch.) Mesfin	Asteraceae	AT00604	Tselime teneg	Herb	Leaf	Eye infection	Squeeze and apply liquid into the affected eye
					Whole	Michi	Crush, filter and drink the fluid.
					Whole	Paralysis	Dry, grind and place powder on fire for fumigation
*Boscia salicifilia* Oliv.	Capparaceae	AT00684	Awo, tetem agajen	Tree	Leaf	Toothache	Chew and hold it on diseased tooth
*Capparis tomentosa* Lam.	Capparaceae	AT00623	Andel	Shrub	Leaf	Evil eye	Place it on fire for fumigation
*Carica papaya* L.	Caricaceae	AT00710	Papaya	Tree	Leaf	Michi	Boil it in water by mixing with leaves of *Eucalyptus globulus* and inhale the vapour
					Latex	Ring worm	Apply latex on affected part
*Carissa spinarum* L.	Apocynaceae	AT00607	Egam	Shrub	Root	Evil eye	Crush it and mix it with whole part of *Withania somnifera* and sulphur and put it on fire for fumigation
					Root	Evil sprit	Crush by mixing with roots of *Clerodendrum myricoides*, *Withania somnifera*, *Jasminum gratissimum* and *Maytenus senegalensis* and place it on fire for fumigation
*Citrus aurantifolia* (Christm.) Swingle	Rutaceae	AT00711	Lemon, lemin	Tree	Fruit	Tinea capitis	Rub it on affected part
					Fruit	Michi	Soak it in water by mixing with leaves of *Rumex nervosus* and whole part of *Withania somnifera* and wash body with it
					Fruit	External wound	Crush by mixing with seeds of *Vicia faba* and apply on affected part
					Fruit	Wound/sore	Rub it on the affected part
*Colutea abyssinica* Kunth & Bouché	Fabaceae	AT00674	Taetaeta	Shrub	Root bark	Evil eye	Tie around the neck
					Root	Toothache	Chew it
*Cordia africana* Lam.	Boraginaceae	AT00683	Awhi	Tree	Leaf	Fire burn	Place it in oven, grind, mix it with butter apply it on affected part
					Leaf	Michi	Crush, filter and drink it alone or by mixing it with boiled coffee
					Leaf	Diarrheal	Crush and drink it alone or by mixing it with boiled coffee
					Leaf	Jaundice	Chew and swallow the fluid in the morning before food
					Leaf	Eye infection	Rub on the affected part
					Leaf	Tonsillitis	Crush, filter and drink the fluid
*Commicarpus pedunculosus* (A.Rich.) Cufod.	Nyctaginaceae	AT00646	Ezni anchiwa	Herb	Leaf	Leshimaniasis	Crush, boil with butter and apply it on affected part
					Root	Dislocated bone	Tie it on the affected part
*Croton macrostachyus* Hochst. ex Delile	Euphorbiaceae	AT00671	Tambok	Tree	Leaf	Jaundice	Boil it in water and drink it alone or with milk
					Latex	Tinea capitis	Apply latex on affected part
					Leaf	Malaria	Boil it in water and drink it with mancheba (milk product)
					Bark	Abdominal pain	Crush with roots of *Cucumis ficifolius*, dry and eat it with butter
*Cynoglossum lanceolatum* Forssk.	Boraginaceae	AT00694 & AT00624	Ni michi, Dekik teneg	Herb	Leaf	Michi	Crush and add the fluid through the ear
							Chew and swallow the fluid
							Place it on fire for fumigation
					Root	Eye infection	Crush and add the fluid into the affected eye
							Boil it in water inhale the vapour
*Cyphostemma oxyphyllum* (A.Rich.) Vollesen	Vitaceae	AT00601 & AT00672	Efchiche, reno	Climber	Root	External wound	Crush and apply it the wound
					Leaf and root	Snake bite	Eat it with honey
							Crush or grind and eat it or drink it with mancheba (milk product)
							Chew and swallow the fluid
							Tie it on the body
*Dovyalis abyssinica* (A.Rich.) Warb.	Flacourtiaceae	AT00656	Mengolhats	Shrub	Fruit	Infection of amoeba, tape worm or ascariasis	Eat the fruit or drink its juice
*Dyschoriste radicans* (Hochst. ex A.rich.) Nees.	Acanthaceae	AT00638	Taetaeta bayta	Herb	Leaf	Anthrax	Crush it by mixing with leaves of *Cucumis ficifolius*, add honey and apply it affected part
					Leaf	Wound/sore	Crush it by mixing with saliva, salt, seeds of *Lepidium sativum* and bulbs of *Allium sativum* and apply or tie on the affected part
*Erythrina abyssinica* Lam. Ex DC.	Fabaceae	AT00602	Zibabeo	Tree	Root bark	Tinea capitis	Crush, mix it with butter and apply on the affected part
*Eucalyptus camaldulensis* Dehnh.	Myrtaceae	AT00673	Keyh kalamites	Tree	Leaf	Eye infection	Boil it with water and inhale its vapour
*Eucalyptus globules* Labill.	Myrtaceae	AT00657	Tseada kalamintos	Tree	Leaf	Eye infection /michi/cough	Boil it with leaves of *Carica papaya* in water and inhale its vapour
					Leaf	Michi	Place it on fire with whole part of *Withania somnifera* and leaves of *Zehneria scabra* for fumigation
							Boil it by mixing with leaves of *Zehneria scabra Cynoglossum lanceolataum* and whole part of *Withania somnifera* in water and inhale its vapour
*Euphorbia* sp.	Euphorbiaceae	AT00697	Tekeze	Herb	Root and leaf	Abdominal pain	Chew and swallow the fluid
					Root	Ascariasis	Chew and swallow the fluid
*Ficus palmata* Forssk.	Moraceae	AT00665	Beles adgi	Shrub	Leaf	External wound	Crush it by mixing with leaves and latex of *Calotropis procera* and apply paste on affected part
					Latex	Ring worm	Apply latex on affected part
					Leaf	Ear diseases	Crush and liquid through the ear
*Ficus vasta* Forssk.	Moraceae	AT00651	Daero	Tree	Bark	Ascariasis	Crush and it with honey
*Hagenia abyssinica* (Bruce) J.F.Gmel.	Rosaceae	AT00714	Habi	Tree	Leaf, fruit and flower	Tape worm	Crush, filter and drink the fluid alone or with milk
*Heliotropium cinerascens* DC. & A.DC.	Boraginaceae	AT00639	Amam gimel	Herb	Leaf	Fire burn	Crush and squeeze liquid onto the damaged part
					Leaf	Tonsillitis	Crush leaves and apply on the shaved head of the sick child
					Leaf	Lip diseases	Crush and spread it on the lips
					Leaf	Michi	Crush, filter and drink the fluid
*Hibiscus micranthus* L.f.	Malvaceae	AT00620	Shigot adgi	Shrub	Leaf	Wound/sore	Crush it by mixing with saliva apply on the wound
*Jasminium gratissimum* Deflers.	Oleaceae	AT00703	Habi tselim	Climber	Root	Evil sprit	Crush by mixing with roots of *Clerodendrum myricoides*, *Withania somnifera*, *Carissa spinarum* and *Maytenus senegalensis* and place it on fire for fumigation
*Kalanchoe quartiniana* A.Rich.	Crassulaceae	AT00693	Adeaka, dekaeta	Herb	Leaf	Paralysis	Crush, add water and wash body with it
*Kniphofia pumila* (Ait.) Kunth.	Asphodelaceae	AT00705	Shingurti zibie	Herb	Bulb	Evil eye	Soak it in water with leaves of *Rumex nervosus* and wash body with it
*Lantana trifolia* L.	Verbenaceae	AT00667	Tsameo	Shrub	Leaf	Ascariasis	Boil it with milk or tea and drink
					Leaf	Amoeba	Boil it in milk by mixing with roots of *Hypoestes forskaolii* and drink
*Maytenus arbutifolia (A.* Rich.) Wilczek	Celastraceae	AT00670	Atat	Shrub	Root	Itching/scabies	Boil it in water and washing body with it
*Maytenus senegalensis* (Lam.) Excell.	Celastraceae	AT00626	Kabkib	Tree	Root	Evil sprit	Crush by mixing it with roots of *Clerodendrum myricoides*, *Withania somnifera*, *Carissa spinarum* and *Jasminum gratissimum* and place it on fire for fumigation
					Leaf	Scorpion bite	Crush, filter and drink the fluid
					Leaf	Tonsillitis	Chew the leaves and spit juice into the mouse of the sick child
					Leaf	Diarrhoea	Crush it, mix it with milk and drink
*Medicago polymorpha* L.	Fabaceae	AT00644	Teneg	Herb	Root	Abdominal pain	Chew and swallow the fluid
*Merendra bengalensis* (Roxb.) Benth.	Lamiaceae	AT00605	Mesaguh	Shrub	Leaf	Ascariasis	Crush, filter and drunk the fluid
					Leaf	Hypertension	Crush, filter and drunk the fluid
*Myrica salicifolia* A.Rich.	Myricaceae	AT00661	Nibie	Tree	Root and bark	Evil eye	Tie it on the body
							Crush and add liquid through the nose
							Tie and place it on fire for fumigation
					Bark	Headache	Crush or grind and add apply liquid through the nose
*Ocimum lamiifolium* Hochst. ex Benth.	Lamiaceae	AT00645	Demekasie	Herb	Leaf	Michi	Boil and inhale the vapour
							Crush and drink it with coffee
*Olea europaea* L. *subsp. cuspidata* (Wall. ex G.Don) Cif., L'Olivicoltore	Oleaceae	AT00663	Awlie	Tree	Leaf	Toothache	Chew it with the affected tooth
					Bark	Malaria	Boil it in water and drink the fluid
					Leaf	Abdominal pain	Chew and swallow the fluid
*Oxalis corniculata* L.	Oxalidaceae	AT00640	Chew mirakut	Herb	Bulb	Tap worm	Peel the external part and eat it alone or mixed with enjera (local food)
*Phytolacca dodecandra* L’Herit.	Phytolaccaceae	AT00662	Shibti	Climber	Whole	abortion	Crush, filter and drink the fluid
					Whole	Abdominal pain/malaria	Crush, filter and drink it with water or locally made beer or milk
					Whole	Bloating	Crush, filter and drink the fluid
*Plantago lanceolata* L.	Plantaginaceae	AT00631	Ni likfti	Herb	Leaf	External wound	Crush and apply it on the affected part
*Polygala abyssinica* Fresen.	Polygalaceae	AT00669	-	Herb	Root	Snake bite, Spider bite	Chew and swallow the fluid
*Rhamnus prinoides* L’Herit.	Rhamnaceae	AT00666	Gesho	Shrub	Seed	Tinea capitis	Rub it on the affected part
					Leaf	Itching/skin rash	Burn it in oven, crush, mix it with butter and apply on the skin
*Rumex abyssinicus* Jacq.	Polygonaceae	AT00692	Mequmeqo	Herb	Leaf and root	Headache	Adding to tea and drunk
					Leaf	Ascariasis	Crush, filter and drink the fluid
					Root	Toothache (tumour)	Crush and drink it with boiled coffee or tea
*Rumex nervosus* Vahl.	Polygonaceae	AT00608	Hehot	Shrub	L & Stem	Ascariasis	Eat or chew and swallow the fluid
					Leaf	Michi	Soak it in water together with whole part of *Withania somnifera* and fruit of *Citrus aurantifolia* and wash body with it
					Leaf	Itching /skin rash	Crush by mixing with leaves of *Withania somnifera*, seeds of *Lepidium sativum* and bulbs of *Allium sativum*, soak it in water and wash body with it
					Leaf	Evil eye	Soak it in water with leaves of *Rumex nervosus* and wash body with it
*Ruta chalepensis* L.	Rutaceae	AT00664	Chena adam	Hoot	Leaf	Evil eye	Crush and drink it with boiled coffee
					Leaf &fruit	Cough	Eat it with food
*Salvia schimperi* Benth.	Lamiaceae	AT00653	Meshendedo	Herb	Leaf	Ring worm	Crush and apply on affected part
*Sarcostemma viminale* (L.) R.Br.	Asclepiadaceae	AT00647	Halengi hibey	Climber	Root	Paralysis	Crush and apply on affected part
*Silene macrosolen* Steud. ex A.Rich.	Caryophyllaceae	AT00691	Saerosaero	Herb	Root	Snake repulsion	Place it on fire for fumigation
					Root	Malaria	Crush and place it on fire for fumigation
*Sorghum bicolor* (L.) Moench.	Poaceae	AT00698	Keyh leqa	Herb	Seed	Herpes zoster	Boil it in water and wash body with it
*Thymus schimperi* Ronniger	Lamiaceae	AT00660	Tesne	Herb	Whole	Toothache	Chew it with the affected tooth
					Whole	Abdominal pain	Boil it with milk and drink
							Chew and swallow the fluid
*Trichodesma trichodesmoides* (Bunge) Gurke.	Boraginaceae	AT00704	Ahimlto	Herb	Leaf	Abdominal pain	Crush and drink the fluid
					Leaf	Bloating	Crush, filter and drink the fluid
*Verbena officinalis* L.	Verbenaceae	AT00619	Atush	Herb	Root	Tonsillitis	Chew it and spit juice into mouse of the sick child
							Crush it, add boiled coffee and drink
							Crush and place it on the shaved head of the sick child
					Leaf	Michi	Place it on fore for fumigation
							Crush, boil, filter and drink the fluid
					Whole	Evil eye	Place it on fire with sulphur for fumigation
					Whole	Abdominal pain	Crush, filter and drink the fluid or chew and swallow the fluid
					Leaf	Ear diseases	Crush, add oil and apply some drops through the ear
					Root	Ascariasis	Crush it by mixing with roots of *Zehneria scabra*, filter and drink the fluid
*Vernonia bipontini* Asch.	Asteraceae	AT00616	Endigendig	Shrub	Root	Child disease that break backbone	Crush, filter and drink the fluid
					Root	Snake bite	Crush, filter and drink the fluid
							Chew and swallow the fluid
					Root	Abdominal pain	Chew and swallow the fluid
*Vicia faba* L.	Fabaceae	AT00702	Ater, alqay	Herb	Seed	Anthrax	Grind and apply the paste
					Seed	External wound	Crush it by mixing with fruits of *Citrus aurantifolia* and apply on the affected part
					Seed	Swelling	Grind it by mixing with seeds of *Trigonella foenum-graecum* and apply it on the affected part
*Zehneria scabra* (L.f.) Sond.	Cucurbitaceae	AT00655	Hafaflo	Climber	Leaf	Paralysis	Crush and tie it on the affected part
					Leaf	Michi	Place it on fire by mixing with whole part of *Withania somnifera* & leaves of *Eucalyptus globulus* for fumigation
					Leaf	External wound	Crush and apply it on the affected part
					Leaf	Eye infection	Boiled it in water by mixing with leaves of *Eucalyptus globulus*, *Withania somnifera*, *Achyranthes aspera* and *Bidens camporum* and inhale the vapour
					Root	Abdominal pain	Chew and swallow the fluid before food
					Root	Ascariasis	Crush by mixing it with *Verbena officinalis*, filter and drink the juice
*Zingiber officinale* Roscoe.	Zingiberaceae	AT00715	Zingible	Herb	Root	Abdominal pain	Chew and swallow the fluid
					Bulb	Vomiting and diarrhea	Chew and swallow the fluid
*Ziziphus spina-christi* (L.) Desf.	Rhamnaceae	AT00622	Geba, kusra	Tree	Leaf	Dandruff	Dry, grind, mix it with butter and rub it on the affected part
					Leaf	Head wound infection	Crush and rub it on the affected part

**Table 3:** Medicinal plants used to treat livestock diseases only

**Scientific name**	**Family name**	**Voucher No.**	**Tigrigna name**	**Habit**	**Part used**	**Used for**	**Preparation and application**
*Buddleja polystachya* Fresen.	Budlejaceae	AT00628	Metere	Shrub	Leaf	Leeches	Crush and add liquid through the nose
*Dregea schimperi* (Decne.) Bullock.	Asclepiadaceae	AT00689	Shanqoq	Climber	Leaf	Rabbis	Crush and drink the fluid
*Leucas abyssinica* (Benth.) Briq.	Lamiaceae	AT00629	Siwa karni	Shrub	Leaf	Eye infection	Chew it and spit juice into the affected eye
					Root	Urine retention	Tie it on the tail
*Lycopersicum esculantum* Mill.	Solanaceae	AT00712	Komodere	Herb	Leaf	Leeches	Crush and add fluid through their nose
*Nicotiana glauca* Graham	Solanaceae	AT00613	Chenawi (tegegwe)	Shrub	Leaf	Lice and ticks infestation (livestock)	Crush by adding water and smear on affected part or wash with it
					Leaf	Leeches infestation (livestock)	Crush, filter and add fluid through the nose
*Nicotiana tabacum* L.	Solanaceae	AT00676	Timbako	Herb	Leaf	Leeches infestation (livestock)	Crush and add fluid through the nose
					Leaf	Scabies and lice infestation (livestock)	Crush, add water and wash with it
*Plumbago zeylanica* L.	Plumbaginaceae	AT00701	Afthi	Herb	Root	Wound (livestock)	Grind and apply on affected part
*Pterolobium stellatum* (Forssk.) Brenan.	Fabaceae	AT00696	Qenteftefe	Shrub	Root	Dislocated bone	Operate the damaged part and put remedy mixed with butter into it

### Diseases treated

The plants were used to treat 47 human and 19 livestock diseases. Of the total medicinal plants, 56% were used to treat human diseases only (Table 1), 37% were used against diseases of both human and domestic animals (Table 2) and 7% were employed to treat diseases of domestic animals only (Table 3). With regard to human diseases, abdominal pain was the one against which a high number of medicinal plants (26 species) were prescribed, followed by wound (21 species), febrile illnesses (19 species), evil eye (19 species), toothache (15 species), ascariasis (15 species), anthrax (14 species), Tinea capitis (12 species), snake bite (10 species), tonsillitis (10 species), eye infection (10 species) and itching (10 species). Preference ranking exercise on six medicinal plants used to treat abdominal pain revealed *Solanum mariginatum* as the most preferred medicinal plant, followed by *Cucumis ficifolius* and *Olea europaea* subsp. *cuspidata* (Table 4).

**Table 4:** Preference ranking to medicinal plants used to treat abdominal pain

**List of medicinal plants**	**Informants**										
	**R**_**1**_	**R**_**2**_	**R**_**3**_	**R**_**4**_	**R**_**5**_	**R**_**6**_	**R**_**7**_	**R**_**8**_	**R**_**9**_	**Total**	**Rank**
*Cucumis ficifolius*	5	6	2	4	2	6	4	6	4	39	2^nd^
*Solanum marginatum*	6	5	4	5	6	4	5	2	6	43	1^st^
*Euclea racemosa* subsp. *schimperi*	2	3	1	3	4	1	6	5	2	27	5^th^
*Abutilon bidentatum*	1	1	3	6	1	2	3	1	1	19	6^th^
*Olea europaea subsp. cuspidata*	4	2	6	2	5	3	1	4	5	32	3^rd^
*Thymus schimperi*	3	4	5	1	3	5	2	3	3	29	4^th^

*Key: Where R represented respondents.

### Plant parts used and modes of remedy preparations

According to interview results, leaf was the most commonly used plant part accounting for 43% of the total reported medicinal plants, followed by roots (26%) and whole part (6%). It was found out that most remedies were processed by crushing (34%), chewing (12%) or boiling (8%) or used in unprocessed form (8%). The majority (59%) of remedies were prepared from fresh materials only. Some remedies were prepared from either dried or fresh materials (30%) while few (11%) were prepared from dried materials only. Water and different additives such as honey, sugar, butter, salt, coffee, tea and milk were often used in the preparation of remedies. The additives were claimed to either reduce poisoning or improve flavour.

### Routes of remedy administration and dosage

More than half (55%) of the remedy preparations were applied externally by spreading them directly on the affected part of the skin, tying or fumigation, and 45% of preparations were applied internally, of which oral was the most commonly used route of application accounting for 36% of the total remedies, followed by local (5%), nasal (3%) and auricular (1%).

Result shows that there was no agreement in measurement or unit used among the informants. Most informants used measuring units such as cup, spoon, drops and fingers but still differed in the doses they administered. Most of the remedies were reported to have no adverse effects except for some species such as *Phytolacca dodecandra*, *Euphorbia abyssinica and Nicotiana glauca* that were indicated to be poisonous both to human and domestic animals.

### Multipurpose medicinal plants

Result of direct matrix ranking conducted by nine key informants on seven selected multipurpose medicinal plants showed *Cordia africana* as the most preferred multipurpose plant, followed by *Eucalyptus globules*, *Opuntia ficus-indica* and *Dodonia angustifolia* (Table 5).

**Table 5:** Direct matrix ranking exercise on seven multiple purpose medicinal plants

**Species use**	***Acacia abyssinica***	***Acacia etbaica***	***Olea europaea subsp. cuspidata***	***Cordia africana***	***Dodonia angustifolia***	***Opuntia ficus-indica***	***Eucalyptus globulus***
Medicine	1	3	5	6	4	2	7
Fire wood	2	3	4	7	5	1	6
Construction	2	3	4	5	6	0	7
Fence	6	7	1	2	3	5	4
Forage	6	4	5	2	3	7	0
Edible fruit	0	0	0	6	0	7	0
Total	17	20	19	28	21	22	24
Rank	7^th^	5^th^	6^th^	1^st^	4^th^	3^rd^	2^nd^

### Marketability of medicinal plants

There were no reports of medicinal plants being sold in open markets solely for their medicinal use*.* But, some medicinal plants were indicated to be sold in local market but for their uses as food, spices and beverages. These include *Allium sativum* (spice), *Carica papaya* (food), *Citrus aurantifolius* (food), *Lepidium sativum* (spice), *Lycopersicum esculantum* (food), *Opuntia ficus-indica* (food), *Rhamnus prinoides* (additive for fermented beverages), *Ruta chalepensis* (spice), *Sorghum bicolor* (food), *Trigonella foenum-graecum* (spice), *Vicia faba* (food), *Zingiber officinale* (spice) and *Ziziphus spina-christi* (food).

### Habitats and conservation status of medicinal plants

Out of the total medicinal plants, 84 (74%) were obtained from wild, 16 (14%) were cultivated in home gardens, and 14 (12%) were either grown in homegardens or harvested from the wild.

According to informants, nowadays search for some medicinal plants, especially trees and some shrubs, required a lot of time and travelling long distances. Of the total reported medicinal plants, 48% were rarely encountered, while 43% were commonly found and the rest (9%) were moderately or occasionally encountered. Result of preference ranking exercise on six medicinal plants, reported by the most informants in the District as threatened species, shows that that *Myrica salicifolia* was among the highly threatened species, followed by *Boscia salicifolia*, *Acokanthera schimperi*, *Acacia abyssinica*, *Olea europaea* subsp. *cuspidata* and *Acacia abyssinica*. The principal threats of medicinal plants in the area were reported to include drought, overgrazing and firewood collection. Informants ranked drought as the most serious threat to medicinal plants followed by overgrazing, firewood collection, agricultural expansion, soil erosion and collection of other different factors.

## Discussion

Despite the large scale environmental degradation and recurrent droughts, there is still rich knowledge on the use of medicinal plants in Kilte Awlaelo District. A total of 114 medicinal plants are in use in the study District to treat various human and animal diseases. Similar studies undertaken in Ofla District, Tigray Region, Ethiopia, came up with 113 medicinal plants [15]. As compared to human diseases, diseases of domestic animals in the District were treated with a relatively fewer number of plant species, which could be due to the less number of diseases affecting the later. Similar findings were reported by studies conducted elsewhere in Ethiopia [16,17]. High number of medicinal plants is used in the treatment of abdominal pain and this may suggest the high importance or prevalence of the disease in the study district. The fact that *Solanum mariginatum* is the most frequently used plant to treat abdominal pain could indicate better efficacy of the plant or its higher abundance in the study District.

Most of the plant species reported were also mentioned by authors in studies conducted elsewhere in Ethiopia; Of the medicinal plants reported by the current study, 59 were mentioned in Abdurhman [15], 50 in Senai [18], 46 in Giday and Ameni [19], 29 each in Getahun [20] and Teklehaymanot and Giday [21], 19 in Mesfin *et al*. [22], 16 in Abebe and Hagos [23], 15 in Yirga [7], 14 in Giday [24] and nine in Ragunathan and Abay [6].

Leaf was the most used plant part in the preparation of remedies in the District as compared to other parts. Many studies conducted elsewhere in Ethiopia also showed the dominance of leaves in the preparation of remedies [8,19,24-27]. In contrast, another study [28] indicated root as the most commonly harvested plant part in a study carried out in five Districts of Tigray Region, Ethiopia. A study conducted in Mana Angetu District, in Oromia Region of Ethiopia also witnessed the common usage of root [29]. Harvesting root of a plant poses more threat to survival of plant than collecting other parts such as fruits, seeds and leaves [6].

According to current results, herbal remedies are largely prepared using fresh materials. There were also many plants from which parts were claimed to either be used as dried or fresh materials. The fact that both forms are used in the preparation of remedies in a given community creates a better opportunity for people to have access to materials used in medicine preparation across the different seasons of the year.

The current study indicated that there was no agreement in measurement or unit used among informants. Most informants reported use of measuring units such as cup, spoon, drops and fingers but still there was difference in doses. The variation in quantity, unit of measurement, and duration of treatment of prescribed plant preparations was also noted in a study conducted elsewhere in the country [28]. A study reported that lack of precision and standardization of preparations are two of the drawbacks of traditional health care system [20].

The greater proportions of remedies were applied externally, which is in agreement with result of a study conducted in Bench District, south-western Ethiopia [30]. However, studies conducted in Mana Angetu District, south-eastern Ethiopia [29] and Konta Special District, Southern Ethiopia [27] revealed that most medicinal plant preparations were taken internally, out of which drinking takes the highest proportion.

Nearly half of the medicinal plants recorded were herbs which may indicate their relatively better abundance as compared to other life forms. Other studies conducted elsewhere in Ethiopia also indicated the dominance of herbs [16,21,27,28]. However, a study conducted in Mana Angetu District, Oromia Region of Ethiopia, reported the dominance of shrubs in medicinal plant preparations [29]. The fact that most of the woody plants in the current study area are depleted could have forced the local people to dwell more on herbaceous medicinal plants. It is not a common practice in the District to sell medicinal plants in local markets, which is in agreement with the finding of study carried out in Bench District, south-western Ethiopia [30].

The majority of medicinal plants in the study District were obtained from the wild. This result agrees with that of other studies conducted elsewhere in the country [14,19,24,26,27,31]. As most of medicinal plants in the District are harvested from the wild, they are highly exposed to various anthropogenic and natural factors and as a result many of them are rarely encountered. Special attention is needed to be given to the medicinal plants that were indicated by preference ranking exercise as the most threatened ones.

There is little practice of cultivating medicinal plants in the area, which is in agreement with other studies conducted elsewhere in the country [15,24,26]. The local community in the study District is not giving much attention for management of medicinal plants. This could be explained by the lack of knowledge among ordinary people about the importance of medicinal plants as most of them are only known by few knowledgeable people.

## Conclusion

Despite the large scale environmental degradation and recurrent drought, medicinal plants are still playing significant role in the management of various human and livestock diseases in Kilte Awulaelo District. In the District, 114 medicinal plants were reported to be used to treat various human and livestock diseases. Relatively higher number of medicinal plants was used in the treatment of human diseases as compared to that used against livestock diseases, which might be attributed to the higher number of diseases affecting the former. Result demonstrated the usage of high number of medicinal plants to treat abdominal pain, probably suggesting high importance or prevalence of the disease in the study District. *Solanum mariginatum* was the most frequently used plant to treat abdominal pain and this could indicate better efficacy or higher abundance of the plant in the study District. Leaf was the most frequently used plant part in the preparation of remedies in the District. Herbs took the higher proportion of the reported medicinal plants, which could be an indication of their relatively better abundance as compared to other life forms. Recurrent drought was reported to have seriously threatened medicinal plant resources in the study area. Despite this fact, there is little effort in the District to cultivate or mange medicinal plants. Thus awareness is needed be raised among local people on sustainable utilization and management of the plant resources. *Ex situ* and *in situ* conservation measures should be taken to protect the medicinal plants of the District from further destruction and special attention should be given to the medicinal plants that were indicated by preference ranking exercise as the most threatened ones.

## Competing interests

The authors declare that they have no competing interests.

## Authors’ contributions

The three authors had significant intellectual contribution towards the design of the study, data collection and analysis and write-up of the manuscript. The authors read and approved the final manuscript.
